# An Integrative Pharmacology-Based Approach for Evaluating the Potential Effects of Purslane Seed in Diabetes Mellitus Treatment Using UHPLC-LTQ-Orbitrap and TCMIP V2.0

**DOI:** 10.3389/fphar.2020.593693

**Published:** 2021-02-02

**Authors:** Jinli Hou, Xiang Zhou, Ping Wang, Chunhui Zhao, Yuewen Qin, Feng Liu, Liping Yu, Haiyu Xu

**Affiliations:** ^1^ Institute of Chinese Materia Medica, China Academy of Chinese Medical Sciences, Beijing, China; ^2^ Medical College, Shaanxi Institute of International Trade and Commerce, Xianyang, China; ^3^ College of Traditional Chinese Medicine, Yunnan University of Chinese Medicine, Kunming, China; ^4^ State Key Laboratory of Innovative Drug and Efficient Energy-Saving Pharmaceutical Equipment, Jiangxi University of Traditional Chinese Medicine, Nanchang, China; ^5^ Guangzhou Zhongda Pharmaceutical Development Co. Ltd., Guangzhou, China

**Keywords:** purslane seed, diabetes mellitus, UHPLC-LTQ-orbitrap, TCMIP v20, molecular network

## Abstract

*Portulaca oleracea L.*, known as the “vegetable for long life,” is an annual succulent herb that is widely distributed worldwide. Many clinical and experimental studies have demonstrated that purslane seed (MCXZ) can be used as an adjunctive and alternative therapy for the treatment of diabetes mellitus (DM). However, the underlying active constituents and pharmacological mechanisms through which MCXZ exerts effects in DM remain unclear. In the present study, we confirmed that MCXZ treatment resulted in hypoglycemic activity, lowering the fasting blood glucose and glycated hemoglobin levels in streptozotocin-induced diabetic mice. Then, ultra-high-pressure liquid chromatography coupled with linear ion trap-Orbitrap tandem mass spectrometry was used to systematically analyze the chemical profile of MCXZ, resulting in the identification of 84 constituents, including 31 organic acids and nine flavonoids. Finally, the Integrative Pharmacology-based Research Platform of Traditional Chinese Medicine was employed to analyze the key active components of MCXZ and the molecular mechanisms through which these components acted in DM. Ten key active compounds were identified based on the topological importance of their corresponding putative targets within the known DM-associated therapeutic target network of known MCXZ putative targets. Functionally, these candidate targets play critical anti-hyperlipidemia, anti-hyperglycemia, immunity regulation, and inflammatory roles involving DM-related pathways, such as the vascular endothelial growth factor (VEGF) signaling pathway and Fc gamma R-mediated phagocytosis, which indicated that MCXZ exhibited anti-diabetic activity through multi-faced actions.

## Introduction

Diabetes mellitus (DM) represents a major public health issue, causing serious economic burdens for both developed and developing countries (Papatheodorou et al., 2018). The International Diabetes Federation (IDF) reported that approximately 463 million individuals had diabetes worldwide in 2019, including 116 million patients in China, which was ranked first in the world (International Diabetes Federation (IDF), 2019). Persistent hyperglycemia and long-term metabolic disorders may lead to the development of nephropathy, retinopathy, neuropathy, and cardiovascular disease (Zhang H. et al., 2018). Currently, the drugs used to treat DM include biguanide, sulfonylureas, α-glycosidase inhibitors, benzoin acid, and derivative secretagogues, most of which aim to control blood glucose levels and must be used long-term. Gradual increases in the required doses of these drugs can lead to liver and kidney dysfunction, which can be associated with various complications, in addition to those resulting from the disease process (Moukette et al., 2017). Therefore, the development of safer, more effective drugs, especially those derived from natural products, which can provide improved management for blood glucose and diabetes-associated complications, has long been the focus of DM studies.

Traditional Chinese medicine (TCM) is practiced as a form of holistic and personalized medicine and has been shown to effectively lower blood glucose levels, control diabetic complications, and cause fewer side-effects than western medicines, based on syndrome differentiation and treatments (Zhang, 2014) that are multi-component, multi-pattern, and multi-target. As a result, increasing research has focused on the TCM-based treatment of DM. In TCM theory, DM belongs to the category of “Xiao-Ke-Zheng,” which was first recorded in the classical medical text “Huangdi Neijing” over a thousand years ago. Purslane is an annual succulent herb best known as the “vegetable for long life” and is distributed throughout diverse geographical environments worldwide. Purslane seed (MCXZ) has been used as both food and medicine for thousands of years in China (Aberoumand, 2009). Clinically, MCXZ, as an adjuvant combined with other treatments, has been shown to alleviate DM symptoms, including reduced inflammation and improved liver function (El-Sayed, 2011; Dehghan et al., 2016). Chemically, a wide variety of compounds have been identified in MCXZ, including flavonoids, polysaccharides, fatty acids, proteins, glutathione, antioxidants, and vitamins (El-Sayed, 2011). Pharmacologically, MCXZ has been associated with various biological activities, including hypoglycemic (A. Mohamed et al., 2019), hypocholesterolemic (Movahedian et al., 2007), anti-oxidative (Guo et al., 2016), diuretic, antipyretic, analgesic, and anti-inflammatory (Daniel, 2006) processes. Currently, MCXZ is used as an adjuvant treatment for DM to improve glucose tolerance, lipid metabolism disorders, liver functions, and insulin sensitivity and reduce hyperinsulinemia (Mohanapriya et al., 2006; El-Sayed, 2011). However, the potential active components and molecular mechanisms through which MCXZ acts and that may be applied to the direct treatment of DM remain unclear, which limits the clinical applications of MCXZ.

**FIGURE 1:**
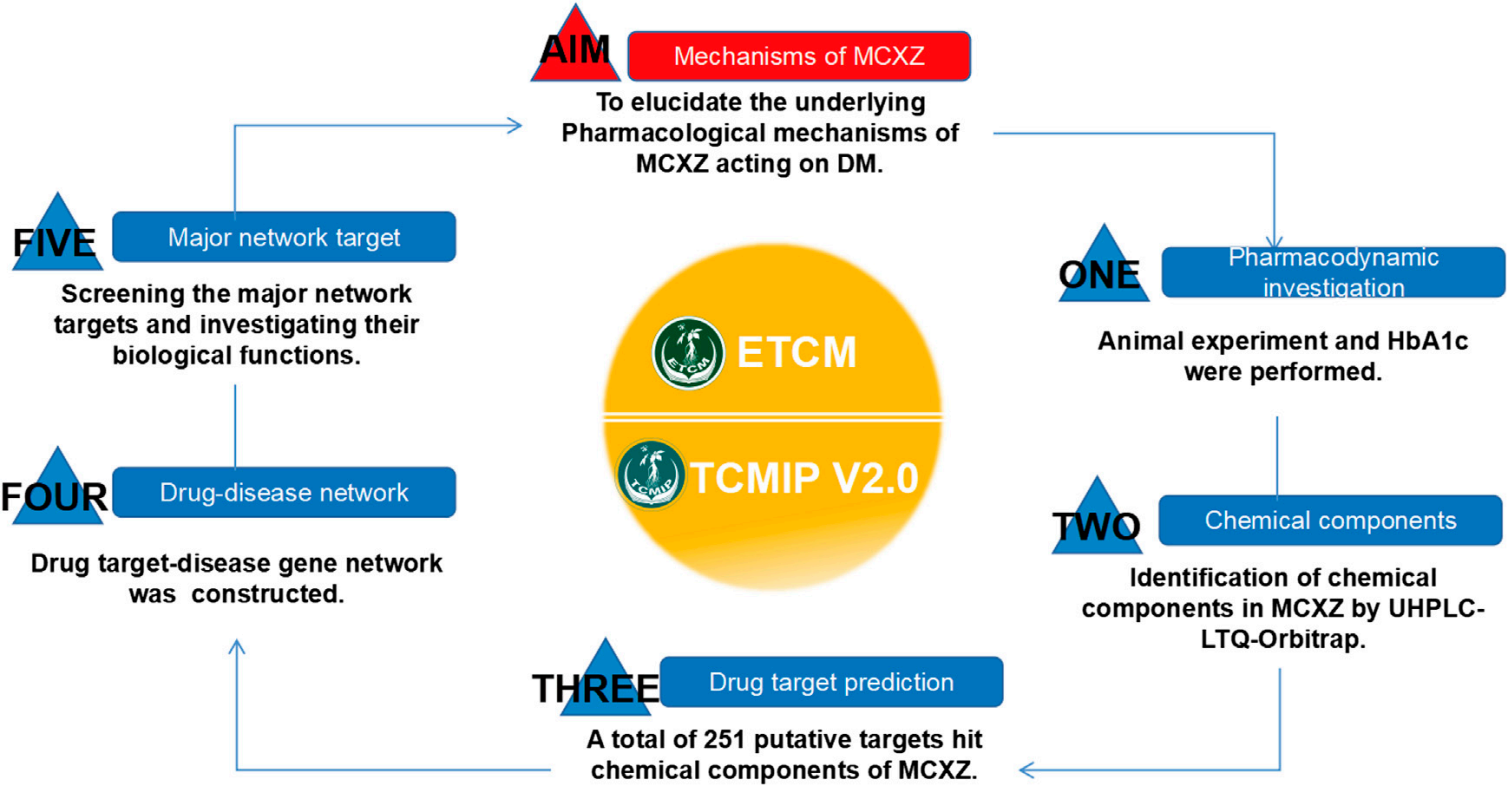
Schematic diagram for revealing the active constituents and the underlying pharmacological mechanisms of MCXZ in the treatment of DM by combining high-throughput technology and integrative pharmacology method.

In the current study, an integrative pharmacology approach was used to investigate the active constituents and the underlying pharmacological mechanisms through which MCXZ acts during the treatment of DM. This study combined high-throughput chemical analysis, target prediction, and network construction and analysis, which was performed by following a three-step analytical process (Figure 1). 1) Chemical information databases, including the Encyclopedia of Traditional Chinese Medicine (ETCM) and other electronic databases, were searched for the constituents of purslane. 2) Ultra-high-pressure liquid chromatography coupled with linear ion trap-Orbitrap tandem mass spectrometry (UHPLC-LTQ-Orbitrap) was performed to rapidly characterize the preliminary chemical profile of MCXZ. 3) The TCMIP V2.0 platform was utilized to predict MCXZ putative targets, construct a drug target-disease-gene network based on predicted interactions among MCXZ putative targets and known therapeutic targets associated with DM-related diseases, and identify potential active constituents correlated with the candidate MCXZ targets during the treatment of DM.

## Materials and Methods

### Chemicals and Materials

Mass spectrometry-grade methanol, acetonitrile, and formic acid were acquired from Fisher Scientific Co. (Loughborough, United Kingdom). Purified water was prepared by a Milli-Q system (Millipore, Billerica, MA, United States). Other reagents used in the experiment were of analytical grade. MCXZ was supplied by Guangzhou Zhongda Pharmaceutical Development Co., Ltd. (Batch NO. 180201; Guangzhou, China). This drug was identified as a dry, mature seed from *Portulaca oleracea* by the pharmacist Yan Jin (research assistant at the China Academy of Chinese Medical Sciences).

### Animals and Experimental Design

Healthy specific-pathogen-free (SPF)-grade male Balb/c mice (body weight: 18.0–21.0 g) were purchased from the Department of Experimental Animal Science, Department of Medicine, Peking University (Beijing, China). The project identification code was 20160010. All animal experiments were approved by the Committee on Animal Care and Use of the Institute of Chinese Materia Medica, China Academy of Chinese Medical Sciences. Before the experiment began, all animals were placed in a standard laboratory environment, during which they were provided free access to food and water. The experiment did not begin until the animals had adapted to the environment for 3 days**.**

Dried MCXZ was ground into a powder with 80 mesh by a high-speed multi-function grinder (JP-500C, Yongkang Jiupin Industry and Trade Co., Ltd.). Before administration, the powder was dissolved in normal saline containing 0.5% carboxymethyl cellulose sodium to prepare 54.17, 108.33, and 216.67 mg/ml suspensions (Liu, 2018).

Streptozotocin (STZ, 60 mg/kg, dissolved in 0.1 M sodium citrate buffer, pH 4.5) was injected intraperitoneally, continuously for 5 days to induce DM in mice (Goodarzi et al., 2019). To establish the normal control group, 12 healthy mice were injected intraperitoneally with an equal volume of sodium citrate buffer. One week after the last injections, the mice were fasted for 5 h, after which blood was collected from the tail vein, and the fasting blood glucose (FBG) levels were measured using a blood glucose monitor (GT-1980. Aikelai Medical Electronics (Pinghu) Co., Ltd.). Mice with FBG levels greater than 11.1 mmol·L^−1^ were considered to be successful DM model mice. All successfully modeled DM mice were randomly divided into five groups according to body weight and FBG: Model (STZ) group, Met (metformin hydrochloride) group, and MCXZ low-, medium-, and high-dose groups (n = 12 for each group). The dose of metformin hydrochloride used was 130 mg/kg body weight (BW)/day; the low, medium, and high doses of MCXZ powder were 812.5, 1,625, and 3,250 mg/kg BW/day, respectively. The normal control group and model group were administered an equivalent volume of 0.9% NaCl. All groups were treated through intragastric administration for four consecutive weeks.

FBG (fasted for 5 h) was detected every 2 weeks for 4 weeks. At the end of the experiment, the mice were fasted for more than 12 h, and then eye blood samples were collected. Blood samples were collected in blank sterile tubes and allowed to coagulate at room temperature for 1 h. Then, whole blood was centrifuged at 3,500 rpm for 15 min. The serum was separated and stored at −80°C for further studies.

### Measurement of Glycated Hemoglobin (HbAlc)

A specific enzyme-linked immunosorbent assay (ELISA) kit (Cusabio, batch number: M03033575) was used to quantify HbAlc from serum samples. This assay employs the competitive inhibition enzyme immunoassay technique. The experiment was performed according to the manufacturer’s instructions.

### Histopathological Evaluation of Liver and Kidney Tissues

The liver and kidney tissues were removed and immersed in 4% formalin for 72 h at 4°C. To analyze the histopathological changes that occurred in the liver and kidney, sections from paraffin-embedded tissues were stained with hematoxylin and eosin and captured under a light microscope (Olympus, BX51, Japan).

### Chemical Information Database for the Compounds Found in Purslane

The chemical compound database information associated with purslane primarily included chemical name, molecular formula, molecular weight, structural formula, and other information. The chemical components associated with purslane were collected from existing databases, using “*Portulaca oleracea*” as the keyword. These databases included the Encyclopedia of Traditional Chinese Medicine (ETCM, http://www.nrc.ac.cn:9090/ETCM/), which contains information about a total of 7,274 herbal ingredients (Xu et al., 2019). Other resources included electronic databases such as PubMed (https://pubmed.ncbi.nlm.nih.gov/, update on 2019) and CNKI (China Journals of Full-text database; https://www.cnki.net/, update on 2019). Detailed information regarding the identified compounds in purslane is presented in Supplementary Table S1.

### Preparation of Sample Solutions

MCXZ was ground into a powder with 40 mesh, and 1 g of powder was accurately weighed. The powder was dissolved in 10 ml 70% methanol and submitted to ultrasonic extraction for 40 min. The extracts were centrifuged for 12 min (at 12,000 rpm), and the supernatant was separated. The sample solution was subjected to 0.22-mm nylon membrane filtration and analyzed directly by UHPLC-LTQ-Orbitrap.

### LC System

Sample analysis was performed using the Thermo Accela UHPLC system (Thermo Fisher Scientific, San Jose, California, United States). Chromatographic separation was performed on a maintained reverse-phase column Waters HSS T3-C18 (2.1 × 100 mm, 1.8 µm). The mobile phase was a mixture of methanol (A) and 0.1% formic acid in water (B). The following elution gradient was used: 0–5 min, 3%–10% A; 5–25 min, 10%–40% A; 25–35 min, 40%–60% A; 35–45 min, 60%–80% A; 45–50 min, 80%–95% A; 50–60 min, 95% A. The flow rate was set to 0.3 ml/min, and the injection volume was 1 µL.

### Mass Spectrometry and Data Processing

For LC-ESI-MS^n^ experiments, the samples were detected in the positive and negative ion modes by electrospray ionization (ESI) source and were scanned in one-stage and multi-stage modes separately. The parameters for the ESI source were as follows: ion source voltage, 3.5 kV; capillary temperature, 350°C; sheath and auxiliary gas pressure, 0.24 and 0.07 MPa, respectively; ion source temperature, 350°C. The sheath and auxiliary gas was nitrogen in both cases. The mass axis of MS was calibrated using an external standard method (the mass error was less than 5 ppm); mass calibration positive ion selection: 74.09643, 3.06037, 195.08465, 262.63612, 524.26496, and 1,022.00341; negative ion selection: 230.10174, 249.15299, and 407.28030. The MS^1^ was fully scanned and acquired in the range of 50–1,500 m/z, with a resolution of 30,000. The MS^2^ uses a data-dependent scan (DDS). The three peaks with the highest abundance were selected for collision-induced dissociation to obtain MS^2^ data.

Mass Frontier 6.0 (Thermo Fisher Scientific) software and Xcalibur 2.1 (Thermo Fisher Scientific) software were employed for data analysis. The accuracy error threshold was fixed at 10 ppm.

### Prediction of Putative MCXZ Targets

According to the results of MCXZ component recognition, the corresponding targets were obtained through target prediction and functional analyses of TCM (including prescriptions) using the Integrative Pharmacology-based Research Platform of Traditional Chinese Medicine (TCMIP V2.0 http://www.tcmip.cn/TCMIP/index.php) (Xu H. Y. et al., 2017). The principle underlying target prediction is the use of MedChem Studio (version 3.0) software to search DrugBank for the structural similarities between the two-dimensional structures of chemical components and the certified drug (Approved), followed by scoring the similarity using the Tanimoto coefficient. When the similarity score was ≥0.8 (moderate-high similarity), the potential targets for the MCXZ chemical components were obtained.

### Prediction of Known Therapeutic Genes Acting on DM

The candidate therapeutic genes associated with DM were collected from the TCMIP V2.0 database using “Diabetes Mellitus” as the keyword. The platform integrates HPO, OMIM, TTD, Drugbank, DisGeNET, ORPHANET, and other drug, biological, and symptom databases.

### Protein–Protein Interaction Data

Protein–protein interactions (PPIs) were obtained by importing putative MCXZ targets and DM-related genes into the STRING database (http://string-db.org/, version 11.0). To ensure the accuracy of the results, the species was set to “Homo sapiens,” and the confidence was set to 0.4.

### Network Analysis and Visualization

To scientifically explain the complex relationships between putative MCXZ targets and known DM-related genes and to identify key nodes, Cytoscape software (version 3.7.1, Boston, MA, United States) was used to create an interaction network between identified components, putative targets, and known DM-related genes. This complex network analysis method includes data integration, analysis, and visualization. The Network Analyzer in Cytoscape software was then used to calculate the three topological parameters of each node gene, including “degree,” “betweenness,” and “closeness.” The core nodes of the interaction network between MCXZ and DM-related targets were obtained by selecting those targets with degree values greater than 2-fold the median value and the key core target network through which MCXZ acts on DM was acquired by selecting nodes that meet all three topological parameters simultaneously. These three topological parameters are typically used to evaluate the topological importance of nodes in molecular interaction networks. The higher the center of a node, the more important that the node was to the network (Mao et al., 2019).

### Pathway Enrichment Analysis

To elucidate the biological functions of putative MCXZ targets, the targets were introduced into DAVID (https://david-d.ncifcrf.gov/, version 6.7), and pathway enrichment analysis was conducted on targets within the network using the Kyoto Encyclopedia of Genes and Genomes database (KEGG, http://www.genome.jp/kegg/). Relevant pathways with *p*-values < 0.05 were selected as significant pathways.

### Quantitative Real-Time Reverse Transcriptase-Polymerase Chain Reaction (qRT-PCR)

Total RNA was isolated from pancreatic tissue using RNAiso Plus (TaKaRa, Tokyo, Japan). The PCR reaction procedures were performed as follows. Stage 1: Pre-denaturation, one cycle at 95°C for 5s. Stage 2: PCR reaction, 40 cycles at 95°C for 10s and 60°C for 30s. Stage 3: 1 cycle heating from 60°C–95°C, at 0.05°C/s. The relative expression levels of vascular endothelial growth factor (*VEGF*), erb-b2 receptor tyrosine kinase 2 (*ErbB2*), androgen receptor (*AR*), and protein kinase B (*Akt1*) were calculated using the 2^−ΔΔCt^ method. β-actin (*ACTB*) was used as the internal control. All quantitative real-time reverse transcriptase-polymerase chain reaction (qRT-PCR) experiments were repeated three times. The primer sequences used in this study were as follows: VEGF-F, 5′-CCT GGG AAA TGT GCC TGT GA-3′ and VEGF-R, 5′-ATT CGC ACA CGG TCT GT-3’; ErbB2-F, 5′-ATT GGC TCT CAT TCA CCG CA-3′ and ErbB2-R, 5′-CCA AGC CCT CAA GAC CAC AT-3’; Akt1-F, 5′-GAT AAC GGA CTT CGG GCT GT-3′ and Akt1-R, 5′-CGG CCA CAC ATC TCG TA-3’; androgen receptor (AR)-F, 5′-GCC CGA ATG CAA AGG TCT TC-3′ and AR-R, 5′-CCC AGA GCT ACC TGC TTC AC-3’; ACTB-F, 5′-AGG GAA ATC GTG CGT GAC AT-3′ and ACTB-R, 5′-AAC CGC TCG TTG CCA ATA GT-3’.

### Statistical Analysis

All data were analyzed by SPSS 25.0 software (SPSS Inc., Chicago, IL, United States). Data were expressed as the mean ± standard error of the mean (SEM). The results were presented using GraphPad Prism 7.0 software (GraphPad Software, San Diego, CA, United States). Significant differences between normally distributed gene expression data were determined by one-way analysis of variance (ANOVA). The FBG and HbA1c data, which were not normally distributed, were analyzed using the nonparametric Kruskal-Wallis test. *p* < 0.05 was considered significant.

## Results

### Effects of MCXZ on FBG and HbAlc Levels in DM Model Mice

**FIGURE 2:**
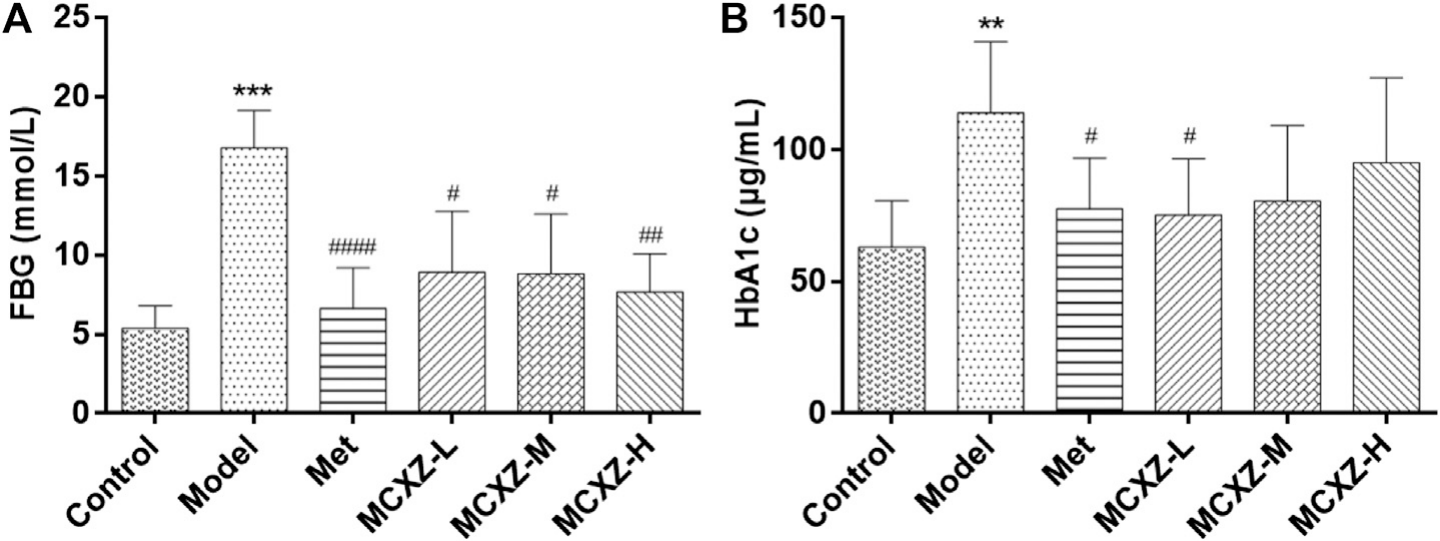
MCXZ alleviates the symptoms of the mice with DM. **(A)** MCXZ reduce the FBG of the mice with DM. **(B)** MCXZ downregulated the level of HbA1c in the serum of mice with DM. MCXZ-L, MCXZ low dose group 812.5 mg/kg; MCXZ-M, MCXZ middle dose group 1625 mg/kg; MCXZ-H, MCXZ high dose group 3250 mg/kg. Data are mean ± SD.***p < 0.001, **p < 0.01 vs. Control; ^####^ *p* < 0.0001, ^##^ *p* < 0.01, ^#^ *p* < 0.05 vs. the modle group; n = 8–12 animals per group.

As shown in Figure 2A, the FBG concentrations were significantly increased in diabetic model mice (model group) compared with those in normal mice (control group), whereas the MCXZ and Met groups showed significantly reduced FBG concentrations compared with that in the model group.

Meanwhile, to further examine the effects of MCXZ on DM, HbAlc levels were detected using an ELISA kit. HbA1c is currently considered the gold standard for glucose monitoring in patients with DM and has been increasingly adopted as a criterion for DM diagnosis. HbA1c levels were substantially increased in the diabetic model mice (model group) compared with those in normal animals (control group). Compared with the model group, mice treated with MCXZ showed significantly decreased HbA1c levels (Figure 2B). Surprisingly, the hypoglycemic effect observed in the low-dose MCXZ group was better than those observed in the medium- and high-dose groups.

### Effects on Liver and Kidney Tissue Histopathology

**FIGURE 3:**
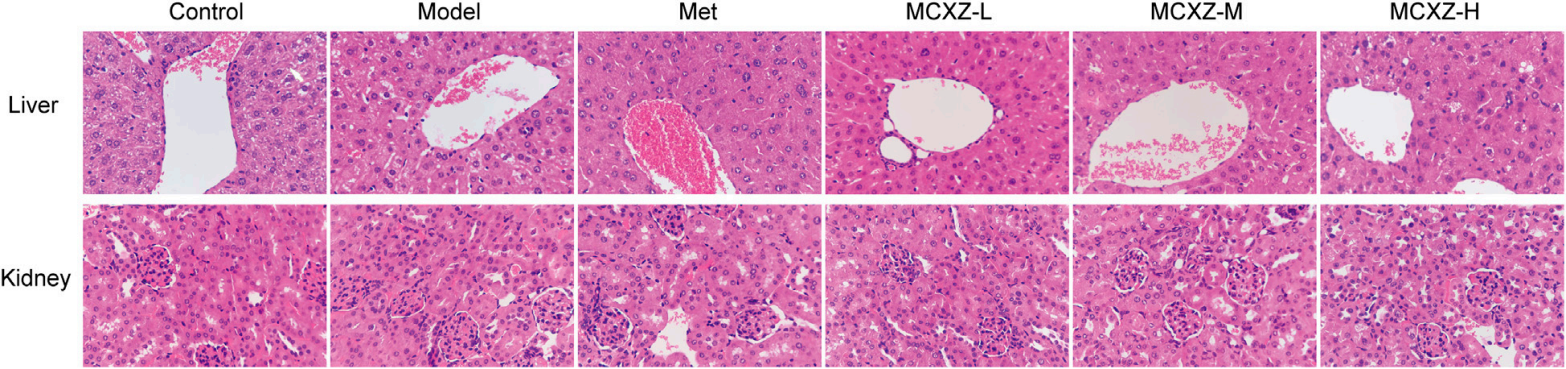
The effects of MCXZ on liver and kidney histopathological injury (× 40).

As shown in Figure 3, compared with the control group, the structures of the liver and kidney tissues were not significantly altered in any of the experimental groups, including the Model, Met, MCXZ-L, MCXZ-M, and MCXZ-H groups.

### Identifification of the Primary Compounds Found in MCXZ Using UHPLC‐LTQ‐Orbitrap

**FIGURE 4:**
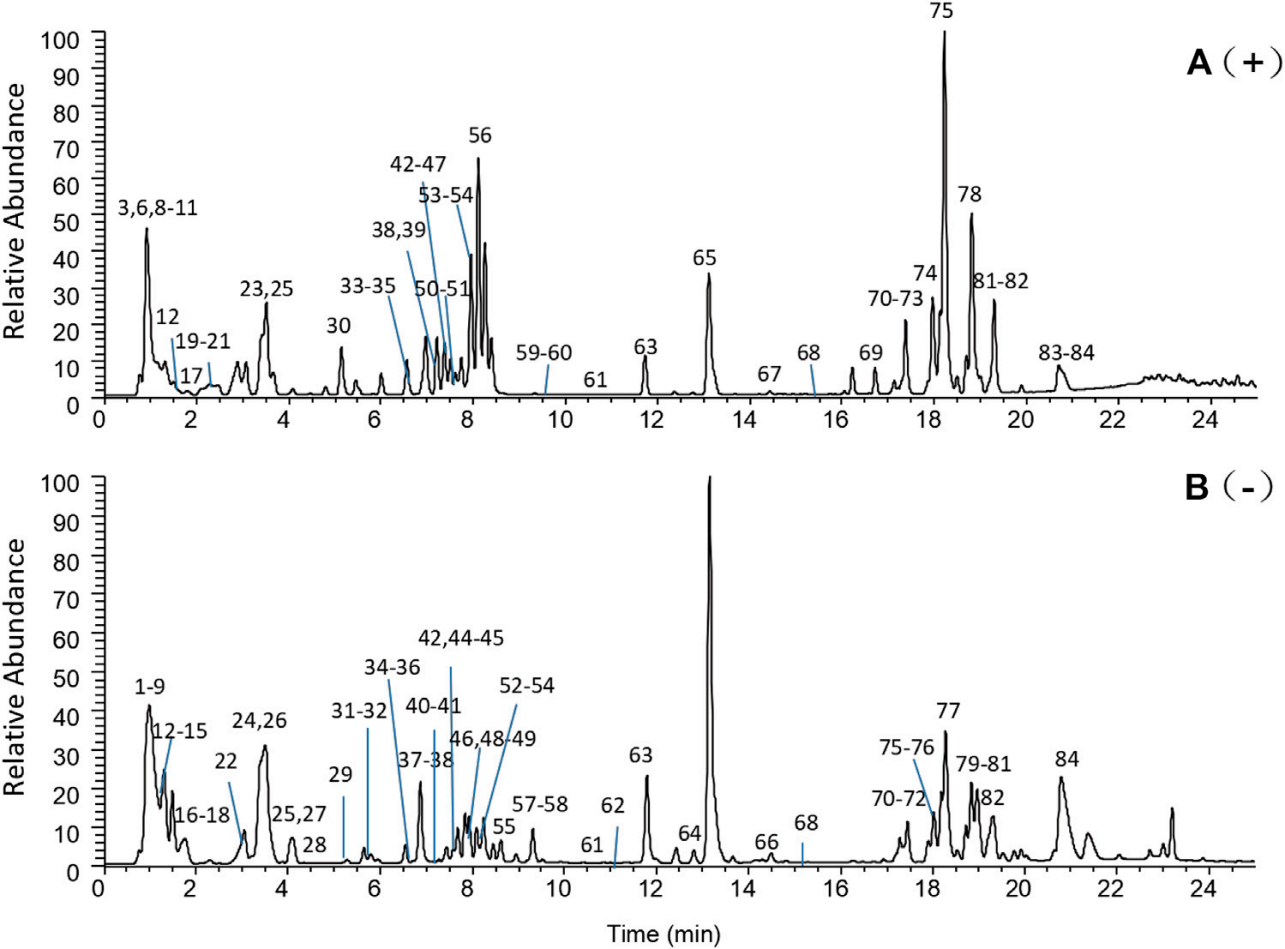
Total ion chromatogram of MCXZ detected by UHPLC-LTQ-Orbitrap. **(A)**: Positive ion detection mode; **(B)**: Negative ion detection mode.

The systemic characterization of chemical profiles is an important precondition for determining the pharmacological mechanisms through which TCM agents exert their effects. To perform this characterization in MCXZ, the UHPLC-LTQ-Orbitrap method, together with the ETCM database, was initially applied for the rapid and high-throughput identification of MCXZ compounds (both known and unknown) in the present study. The UHPLC-LTQ-Orbitrap method combines efficient separation and strong structural characterization abilities to achieve the high-resolution acquisition of parent and daughter ion data, both quickly and simultaneously, to obtain multi-stage mass spectrometry fragment information, which can significantly improve the ability to rapidly identify and analyze the chemical components of complex systems, such as those used in TCM (Wang et al., 2015). The ETCM contains 7,274 herbal ingredients. Any identified molecular formulas that are not included in the purslane chemical components database may represent either known compounds that have not previously been associated with purslane or new compounds; compounds can be searched in his database and confirmed against various types of information. The total ion chromatograms (TIC) of MCXZ were presented in Figure 4, corresponding to the positive and negative signals.

During the identification process, the compounds were first analyzed and identified in positive ion mode, and then further analyzed and verified in negative ion mode. Compounds in MCXZ were identified or tentatively characterized according to their retention times and MS^n^ data, which are summarized in Table 1. The specific method used Xcalibur 2.1 to extract molecular ion peaks from first-order, high-resolution, mass spectrometry data, which were then matched with the high-precision excimer ions in the purslane chemical compound database (the collected compounds were calculated by [M-H]^−^, [M + CH3COO]^−^, [M]^+^, [M + H]^+^, and [M + Na]^+^]. All possible compounds were obtained with a mass error of 5.0 ppm. The MS^n^ information could also be compared against the precise relative molecular mass, fragmentation patterns, and pathways reported in the related literature to identify compounds (Sun et al., 2014; Yu et al., 2016). Using the described sample treatment methods and analytical conditions, a total of 84 compounds were analyzed and identified in MCXZ using both positive and negative ion modes, including 31 organic acids, 22 alkaloids, nine flavonoids, eight coumarins, et al.

**TABLE 1:** Identification of chemical compounds in MCXZ by UHPLC-LTQ-Orbitrap.

Peak no	tR min	Measured mass m/z	MS2	Formula	Compound Name	References	Error ppm
1	0.88	259.0218[M + HCOO]^−^	NO	C_13_H_26_O_2_	Tridecylic acid	Jin et al. (2015)	0.772
2	0.89	214.0480[M-H]^−^	NO	C_12_H_9_NO_3_	Robustine	Xiang et al. (2007)	1.215
3	0.92	146.0458[M-H]^−^	119.0446[M-C_2_H_3_-H]^−^, 102.0350[M-C_2_H_3_-NH_3_-H]^−^	C_5_H_9_NO_4_	L-Glutamic acid	Jin et al. (2016)	0.137
	0.94	148.0610[M + H]^+^	NO				−0.270
4	0.93	193.0352[M + HCOO]^−^	NO	C_5_H_8_O_5_	Malicacid 1-Meester/Malicacid 4-Meester	Jin et al. (2016)	−0.052
5	0.95	185.0220[M + HCOO]^−^	NO	C_6_H_4_O_4_	Coumalic acid	Xiang et al. (2007)	−0.378
6	0.91	116.0346[M + H]^+^	NO	C_4_H_5_NO_3_	5-Hydroxy-2-pyridinecarboxylicacid	Ding et al. (2009)	−0.172
	0.96	114.0310[M-H]^−^	NO				−1.228
7	0.98	205.0348[M-H]^−^	NO	C_7_H_10_O_7_	L-6-citric acid acetate/L-methyl citrate	Jin et al. (2015)	2.341
8	0.98	218.0672[M-H]^−^	NO	C_12_H_13_NO_3_	Oleracein E	Xiang et al. (2005)	0.780
	0.99	242.0783[M + Na]^+^	NO				−0.041
9	0.99	157.0368[M-H]^−^	NO	C_4_H_6_N_4_O_3_	Allantoin	Xiang et al. (2007)	−0.764
		159.0518[M + H]^+^	NO				−0.189
10	0.99	170.0816[M + H]^+^	NO	C_8_H_11_NO_3_	Noradrenaline	Chen et al. (2003)	0.176
11	1.01	163.0605[M + H]^+^	132.0815[M-CH_2_OH+H]^+^, 106.0655[M-CH_2_OH-C_2_H_2_+H]^+^	C_6_H_10_O_5_	Dimethyl malate	Jin et al. (2016)	0.184
12	1.00	133.0142[M-H]^−^	NO	C_4_H_6_O_5_	Malic acid	Oliveira et al. (2009)	1.729
	1.56	135.0281[M + H]^+^	NO				−1.555
13	1.01	115.0035[M-H]^−^	NO	C_4_H_4_O_4_	Fumaric acid	Jin et al. (2015)	2.696
14	1.02	664.2009[M-H]^−^	NO	C_30_H_35_NO_16_	Oleracein C	Xiang et al. (2005)	2.710
15	1.06	191.0193[M-H]^−^	NO	C_6_H_8_O_7_	Citric acid	Jin et al. (2016)	2.513
16	1.60	306.0758[M-H]^−^	NO	C_10_H_17_N_3_O_6_S	Glutathiose	Ying et al. (2018)	0.229
17	1.77	196.0616[M-H]^−^	NO	C_9_H_11_NO_4_	Levodopa	Chen et al. (2003)	−2.040
	1.81	198.0768[M + H]^+^	NO				0.353
18	1.85	279.0692[M + HCOO]^−^	261.0921[M-H_2_O + HCOO]^−^, 233.0973[M-H_2_O-CO + HCOO]^−^, 210.0386[M-H_2_O-CO-Na + HCOO]^−^	C_9_H_14_O_7_	L-1,5-Dimethyl citric acid	Jin et al. (2016)	1.792
19	2.20	138.0195[M + H]^+^	NO	C_8_H_11_NO	Tyramin	Xin et al. (2009)	−0.072
20	2.27	117.0192[M-H]^−^	NO	C_4_H_6_O_4_	Butanedioic acid	Xiang et al. (2007)	−0.085
21	2.29	132.1024[M + H]^+^	NO	C_6_H_13_NO_2_	L-Isoleucine	Jin et al. (2016)	0.151
22	3.07	870.2463[M-H]^−^	NO	C_41_H_45_NO_20_	Oleracein O	Jiao et al. (2015)	0.954
23	3.47	153.0412[M + H]^+^	NO	C_8_H_11_NO_2_	Dopamine	Chen et al. (2003)	1.111
24	3.51	151.0398[M-H]^−^	NO	C_8_H_8_O_3_	4-Hydroxyphenyl acetate/Vanillin	Yue et al. (2015)	−0.265
25	3.52	133.0501[M + H]^+^	NO	C_5_H_8_O_4_	Mono-Methyl succinate	Jin et al. (2016)	0.075
	3.94	131.0349[M-H]^−^	113.9263[M-H_2_O-H]^−^, 104.0254[M-C_2_H_3_-H]^−^, 87.0451[M-C_2_H_3_-NH_3_-H]^−^, 77.0145[M-C_2_H_3_-C_2_H_3_-H]^−^				−0.839
26	3.52	567.1428[M-H]^−^	521.1512[M-C_2_H_5_OH-H]^−^, 506.3255[M-C_2_H_5_OH-CH_3_-H]^−^	C_25_H_28_O_15_	Portuloside B	Seo et al. (2004)	0.141
27	4.10	218.1029[M-H]^−^	NO	C_12_H_13_NO_3_	Trollisine	Yao et al. (2007)	0.504
28	4.67	153.0190[M-H]^−^	109.0296[M-COO-H]^−^	C_7_H_6_O_4_	Protocatechuate	Jiang et al. (2012)	0.850
29	5.21	261.0404[M + HCOO]^−^	NO	C_12_H_8_O_4_	5-Methoxypsoralen	Xiang et al. (2007)	−0.115
30	5.52	162.0555[M + H]^+^	NO	C_9_H_7_NO_2_	Indole-3-carboxylic acid	Yan et al. (2012)	0.370
31	5.72	609.2019[M-H]^−^	NO	C_28_H_34_O_15_	Hesperidin	Yang et al. (2007)	−0.295
32	5.80	137.0242[M-H]^−^	93.0345[M-CO_2_-H]^−^	C_7_H_6_O_3_	Salicylic acid	Gao et al. (2019)	0.438
33	6.37	261.1585[M + H]^+^	243.2121[M-H_2_O + H]^+^, 217.1053[M-H_2_O-C_2_H_2_+H]^+^	C_15_H_20_N_2_O_2_	3-(2-Methylpropyl)-6-benzyl-2,5-diketopiperazine	Ding et al. (2009)	0.038
34	6.43	327.0091[M + Na]^+^	NO	C_18_H_28_N_2_O_2_	Oleracone A	Li et al. (2016)	2.049
		349.1129[M + HCOO]^−^	331.1892[M-H_2_O + HCOO]^−^, 313.0924[M-2H_2_O + HCOO]^−^, 267.0722[M-2H_2_O-NO_2_+HCOO]^−^, 249.0616[M-3H_2_O-NO_2_+HCOO]^−^				−0.057
35	6.59	165.0553[M + H]^+^	NO	C_9_H_8_O_3_	P-Coumaric acid	Xin et al. (2009)	−0.848
	6.74	163.0430[M-H]^−^	136.0516[M-C_2_H_3_-H]^−^, 121.0520[M-C_2_H_3_-CH_3_-H]^−^, 106.0413[M-C_2_H_3_-2CH_3_-H]^−^				0.368
36	6.85	199.0607[M + HCOO]^−^	NO	C_8_H_10_O_3_	3,4-Dihydroxyphenylethanol	Jin et al. (2016)	−0.603
37	6.94	141.0190[M-H]^−^	NO	C_6_H_6_O_4_	3-Hydroxy-5-methylfuran-3-carboxylic acid/5-Hydroxymethyl-2-furoic acid	Jin et al. (2016)	1.276
38	7.13	123.0446[M + H]^+^	95.0494[M-CO+H]^+^, 79.0543[M-CO-O+H]^+^	C_7_H_6_O_2_	P-Hydroxybenzaldehyde	Shan et al. (2015)	−1.057
		121.0294[M-H]^−^	93.0301[M-CO-H]^−^, 77.0396[M-CO-O-H]^−^				−0.165
39	7.22	476.3088[M + Na]^+^	459.2825[M-NH_3_+Na]^+^, 335.2599[M-NH_3_-6H_2_O-NH_2_+Na]^+^	C_24_H_39_NO_7_	Oleraciamide C	Xu L. et al. (2017)	0.336
40	7.22	321.1542[M + HCOO]^−^	NO	C_15_H_20_N_2_O_3_	Cyclotyrosine-leucine	Jin et al. (2016)	0.685
41	7.22	177.0191[M-H]^−^	NO	C_9_H_6_O_4_	6,7-Dihydroxycoumarin	Xiang et al. (2007)	−0.565
42	7.51	548.3380[M + H]^+^	NO	C_26_H_29_NO_12_	Oleracein F	Liu et al. (2011)	1.240
	7.54	546.1013[M-H]^−^	NO				−4.669
43	7.60	534.2263[M + H]^+^	NO	C_25_H_27_NO_12_	Oleracein B	Xiang et al. (2005)	−0.431
44	7.59	502.1347[M-H]^−^	NO	C_24_H_25_NO_11_	Oleracein A	Xiang et al. (2005)	−0.956
	7.62	504.1519[M + H]^+^	NO				0.754
45	7.64	139.1122[M + H]^+^	NO	C_9_H_14_O	2,4-Nonadienal	Zhao et al. (2014)	0.719
		183.1022[M + HCOO]^−^	NO				0.928
46	7.64	287.0562[M + H]^+^	258.0768[M-CO]^+^, 153.0413[M-C_8_H_6_O_2_+H]^+^, 93.9636[M-C_8_H_6_O_4_+H]^+^	C_15_H_10_O_6_	Kaempferol	Yang (2016): Wu et al. (2019a)	0.348
	7.83	285.0409[M-H]^−^	267.1966[M-H_2_O-CO-H]^−^, 239.2017[M-H_2_O-CO-H]^−^, 221.1911[M-2H_2_O-H]^−^				0.070
47	7.73	174.1281[M + H]^+^	NO	C_10_H_7_NO_2_	3-Quinoline carboxylic acid	Yan et al. (2012)	1.321
		172.0227[M-H]^−^	NO				1.337
48	7.83	274.0694[M + HCOO]^−^	255.1966[M -H_2_O + HCOO]^−^, 227.2016[M -H_2_O-CO + HCOO]^−^	C_13_H_11_NO_3_	5,6-Dihydro-8,9-dihydroxy-11H-pyrrolo[2,1-b]benzazepin-11-one	Yue et al. (2015)	−0.109
49	7.92	179.0345[M-H]^−^	135.0452[M-CO_2_-H]^−^, 107.0145[M-CO_2_-H]^−^	C_9_H_8_O_4_	Caffeicacid	Qin et al. (2018); Zhang X. et al. (2018)	−0.114
50	7.92	197.1178[M + H]^+^	219.1754[M + Na]^+^	C_11_H_16_O_3_	Epiloliolide	Jin et al. (2016)	1.979
51	7.94	169.0503[M + H]^+^	141.9829[M-CO+H]^+^, 110.0603[M-CH_3_-COO+H]^+^	C_8_H_8_O_4_	Vanillic acid	Xiang et al. (2007)	−0.237
52	7.97	245.0928[M-H]^−^	NO	C_13_H_10_O_5_	Isopimpinellin	Xiang et al. (2007)	0.898
53	8.00	195.0660[M + H]^+^	177.1284[M-H_2_O + H]^+^, 159.1178[M-2H_2_O + H]^+^, 149.1333[M-H_2_O-CO + H]^+^	C_10_H_10_O_4_	Ferulic Acid	Qin et al. (2018); Zhang X. et al. (2018)	−0.205
		193.0503[M-H]-	178.0635[M-CH_3_-H]^−^, 149.0608[M-COO-H]^−^, 134.0376[M-COO-CH_3_-H]^−^				0.673
54	8.01	193.0501[M + H]^+^	NO	C_10_H_8_O_4_	Scopoletin	Xiang et al. (2007)	0.622
		237.0800[M + HCOO]^−^	NO				−0.127
55	8.11	396.8029[M + H]^+^	381.0306[M-CH_3_+H]^+^	C_26_H_52_O_2_	Cerotic acid	Zheng et al. (2010)	0.126
			368.2269[M-CO_2_+H]^+^				
56	8.35	197.1182[M + Na]^+^	NO	C_9_H_18_O_3_	9-Hydroxynonanoic acid	Zhao et al. (2014)	2.384
57	8.46	479.1186[M + HCOO]^−^	NO	C_26_H_26_O_6_	Lonchocarpicacid	Xiang et al. (2007)	0.167
58	8.97	137.0244[M-H]^−^	108.0452[M-CHO-H]^−^, 92.0268[M-O-H]^−^	C_7_H_6_O_3_	Protocatechualdehyde	Jiang et al. (2012)	−1.241
59	9.54	314.1399[M + H]^+^	NO	C_18_H_19_NO_4_	N-Trans-Feruloyltyramine	Yan et al. (2012)	−0.032
60	9.54	137.1330[M + H]^+^	NO	C_10_H_16_	7-Propylidene-bicyclo[4,1,0]heptane	Zhao et al., 2014	−0.802
61	10.63	333.2284[M + H]^+^	315.2539[M-H_2_O + H]^+^, 297.2432[M-2H_2_O + H]^+^, 279.2326[M-3H_2_O + H]^+^	C_17_H_32_O_6_	3s-3-O-*β*-D-Glucopyranosyl-3,7-dimethyl-octyl-1,6-diene-3-ol	Seo et al. (2004)	−0.030
	10.70	331.2115[M-H]^−^	313.2018[M-H_2_O-H]^−^, 295.1917[M-2H_2_O-H]^−^, 277.1807[M-3H_2_O-H]^−^				−0.030
62	11.04	347.2430[M + HCOO]^−^	NO	C_17_H_18_O_5_	Portulacanone D	Yan et al. (2012)	0.115
63	11.74	351.2155[M + Na]^+^	333.2048[M-H_2_O + Na]^+^	C_22_H_32_O_2_	4,7,10,13,16,19-Docosahexenoic acid(DHA)	Zou (2004)	0.399
	11.80	327.2167[M-H]^−^	309.2075[M-H_2_O-H]^−^, 291.1973[M-2H_2_O-H]^−^, 265.2177[M-2H_2_O-C_2_H_2_-H]^−^, 247.2073[M-3H_2_O-C_2_H_2_-H]^−^				−0.275
64	12.82	331.2480[M-H]^−^	313.2385[M-H_2_O-H]^−^	C_18_H_20_O_6_	Portulacanone C	Yan et al. (2012)	−0.604
65	13.11	395.3155[M + Na]^+^	NO	C_17_H_24_O_9_	Syringin	Gao et al. (2012)	−0.025
66	14.53	269.1303[M-H]^−^	241.0504[M-CO-H]^−^, 240.0434[M-CHO-H]^−^, 225.0555[M-CO_2_-H]^−^, 197.1915[M-CO_2_-CO-H]^−^	C_15_H_10_O_5_	Genistein	Zhao et al. (2019)	−0.047
67	14.69	228.1967[M]^+^	199.1488[M-C_2_H_5_]^+^, 171.1023[M-C_2_H_5_-CO]^+^	C_14_H_28_O_2_	Myristic acid	Li et al. (2008)	−0.131
68	15.67	255.0659[M + H]^+^	227.1804[M-CO+H]^+^, 199.1702[M-2CO+H]^+^	C_15_H_10_O_4_	Daidzein	Fang et al. (2013)	−0.118
	15.17	253.0862[M-H]^−^	209.8848[M-CO_2_-H]^−^, 197.0606[M-2CO-H]^−^				−0.198
69	16.79	303.3095[M + H]^+^	NO	C_20_H_30_O_2_	5,8,11,14,17-Eicosapentaenoic acid (EPA)	Zou (2004)	0.824
70	17.22	433.3270[M + H]^+^	NO	C_21_H_20_O_10_	Genistin	Fang et al. (2013)	−0.231
	17.26	431.3099[M-H]^−^	269.0879[M-Glu-H]^−^				0.023
71	17.39	518.3262[M + H]^+^	NO	C_25_H_27_NO_11_	Oleracein G	Liu et al. (2011)	0.058
	17.46	562.3136[M + HCOO]^−^	NO				0.000
72	17.54	301.1071[M + H]^+^	283.0696[M-H_2_O + H]^+^, 255.1755[M-H_2_O-CO + H]^+^, 239.0800[M-H_2_O-CO-NH_2_+H]^+^	C_17_H_16_O_5_	2,2′-Dihydroxy-4′,6′-dimethoxychalcone	Yan et al. (2012)	3.387
	17.60	299.0764[M-H]^−^	281.2491[M-H_2_O-H]^−^, 253.2541[M-H_2_O-CO-H]^−^				−0.167
73	17.64	330.3014[M + H]^+^	NO	C_18_H_19_NO_5_	N-Trans-Feruloyloctopamine/	Yan et al. (2012)	0.061
					N-Cis-Feruloyloctopamine		
74	17.90	696.2141[M + H]^+^	NO	C_31_H_37_NO_17_	Oleracein D	Xiang et al. (2005)	−1.030
75	17.95	577.2679[M-H]^−^	NO	C_27_H_30_O_14_	Apigenin-4′-O-*α*-L-rhamnopyranoside	Jin et al. (2015)	−0.987
	18.25	579.3233[M + H]^+^	NO				1.484
76	18.19	279.2328[M-H]^−^	261.2222[M-H_2_O-H]^−^, 243.0621[M-2H_2_O-H]^−^	C_18_H_32_O_2_	Linoleic acid	Dong et al. (2020)	−1.361
77	18.64	391.2594[M + HCOO]^−^	373.2502[M-H_2_O + HCOO]^−^	C_19_H_22_O_6_	Portulacanone B	Yan et al. (2012)	0.077
78	18.70	449.2000[M + H]^+^	NO	C_27_H_28_O_6_	Lonchocarpenin	Xiang et al. (2007)	−0.111
79	18.97	255.2322[M-H]^−^	NO	C_16_H_32_O_2_	Palmitic acid	Dong et al. (2020)	0.627
80	18.98	329.2689[M-H]^−^	311.223[M-H_2_O-H]^−^, 293.2122[M-2H_2_O-H]^−^, 275.2015[M-3H_2_O-H]^−^, 229.1449[M-3H_2_O-NO_2_-H]^−^, 211.1344[M-4H_2_O-NO_2_-H]^−^	C_19_H_38_O_4_	Monopalmitin	Qiao et al. (2012)	0.061
81	18.93	307.2639[M + Na]^+^	NO	C_18_H_36_O_2_	Stearic acid	Jin et al. (2016); Zhao et al. (2014)	0.456
	18.98	329.2689[M + HCOO]^−^	311.223[M-H_2_O + HCOO]^−^, 293.2122[M-2H_2_O + HCOO]^−^, 275.2015[M-3H_2_O + HCOO]^−^				0.061
82	19.26	279.2328[M + H]^+^	261.2225[M-H_2_O + H]^+^, 243.2116[M-2H_2_O + H]^+^	C_18_H_30_O_2_	*α*-Linolenic acid	Xin et al. (2008)	0.072
	19.30	323.2216[M + HCOO]^−^	NO				0.062
83	20.36	427.3901[M + H]^+^	408.211[M-H_2_O]^+^	C_30_H_50_O	Lupeol	Li et al. (2014)	0.374
84	20.69	281.2479[M-H]^−^	NO	C_18_H_34_O_2_	Oleic acid/14-Octadecenoic acid	Zhao et al. (2014)	0.533
	20.73	283.2643[M + H]^+^	265.2537[M-H_2_O + H]^+^, 247.2429[M-2H_2_O + H]^+^				−0.282

**FIGURE 5:**
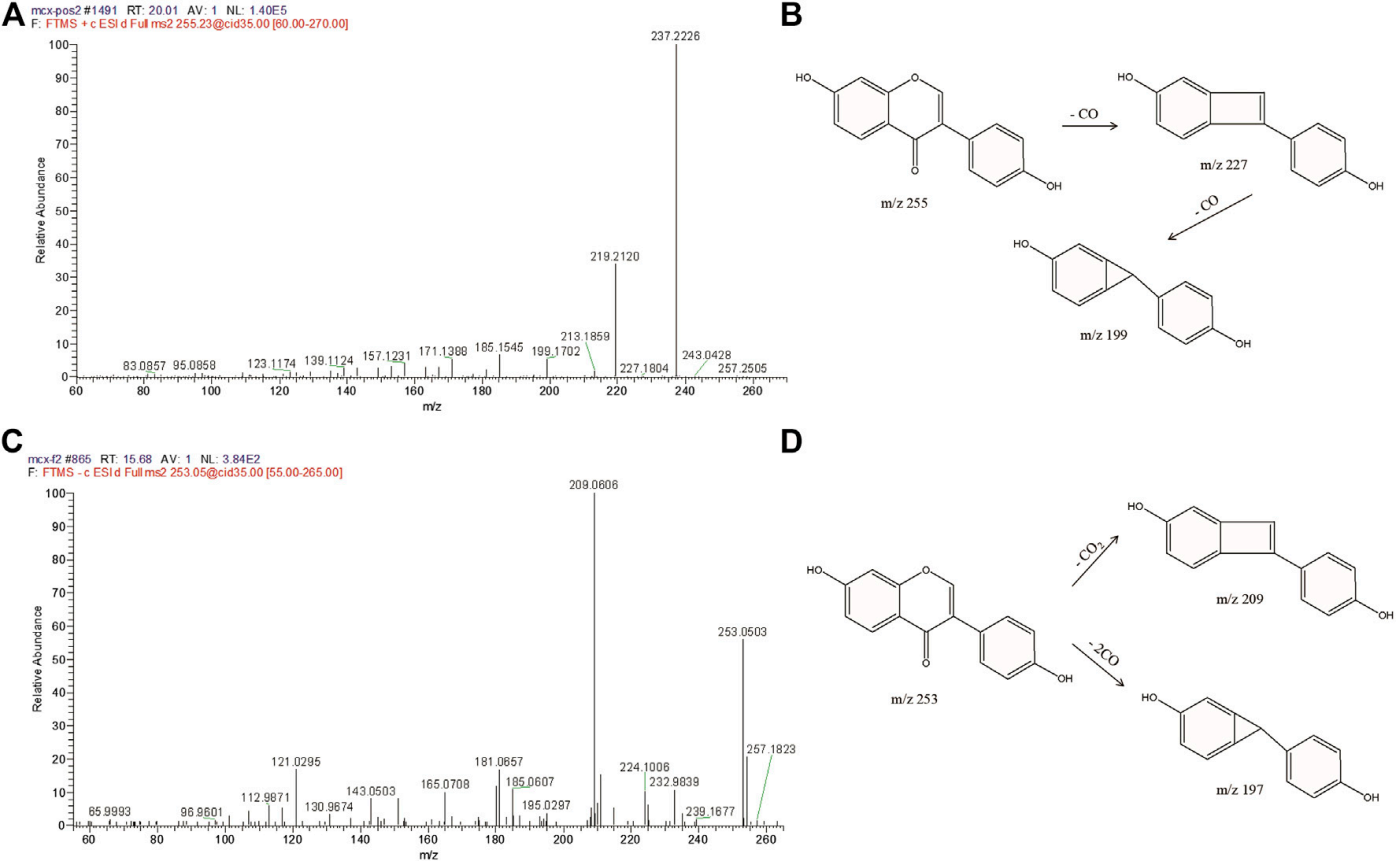
MS^2^ spectrum of Flavonoids and main mass spectrometry fragmentation pathways. MS^2^ spectrum and fragmentation pathways of daidzein in positive ion mode **(A) (B)**. MS^2^ spectrum and fragmentation pathways of daidzein in negative ion mode **(C) (D)**.

For example, flavonoids respond in both positive and negative ion modes, and molecular ions exist in (M + H)^+^, (M + Na)^+^, and (M-H)^−^ form during primary mass spectrometry. High-precision quasi-molecular ions for Compound 68 were obtained in both positive and negative ion modes, at m/z 255.0659 and 253.0862, respectively, which was identified as daidzein (C_15_H_10_O_4_) based on comparisons with the chemical composition database. Meanwhile, in positive ion mode, the MS^n^ information showed that the (M + H)^+^ ion of daidzein at m/z 255 was fragmented by the continuous loss of 28 Da (-CO) at m/z 227 and 56 Da (-2CO) at m/z 199 (Figures 5A,B). In negative ion mode, the MS^n^ information showed that the (M-H)^−^ ion of daidzein at m/z 253 was fragmented by the loss of 44 Da (-CO_2_)at m/z 209 and 56 Da (-2CO) at m/z 197 (Figures 5C and 5D), which was consistent with the results of previous studies (Fang et al., 2013). The above fragmentation pathways provide insights that enable us to speculate on the structures of other flavonoids and their derivatives, such as genistein and kaempferol.

### Putative Targets and the Potential Molecular Basis of the Traditional Efficacy Observed During MCXZ Treatment

The TCMIP V2.0 integrated the ETCM database with a series of authoritative algorithms, including the calculation of physicochemical properties, target prediction, network analysis, and visualization. The TCMIP V2.0 had been used as a powerful platform to construct multi-dimensional correlations for in-depth studies of the molecular mechanisms of TCM (Xu H. Y. et al., 2017). In this study, TCMIP V2.0 was used to perform target prediction for MCXZ.

**FIGURE 6:**
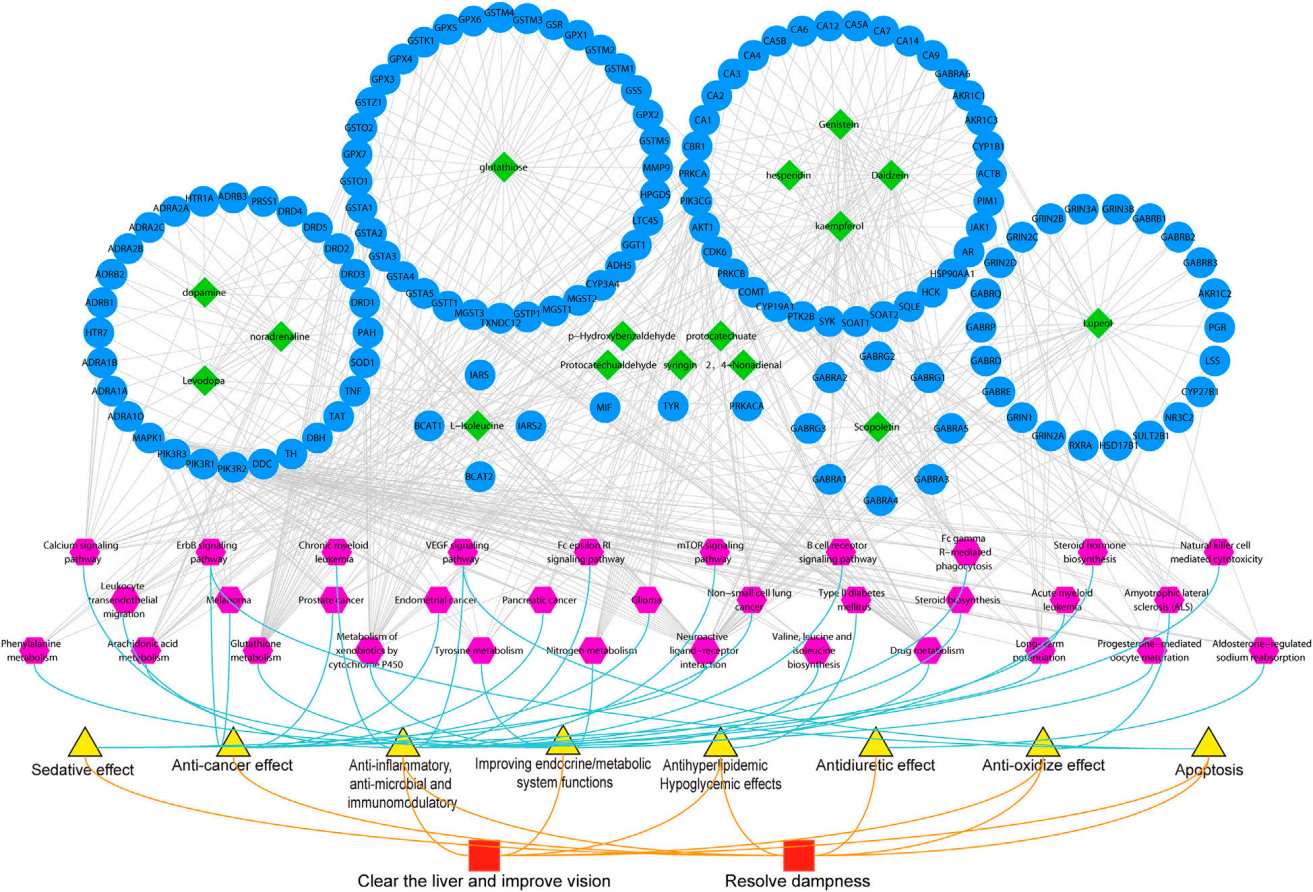
Explain the correlation among the chemical components of MCXZ and the therapeutic efficacy of TCM theory was analyzed based on network pharmacology. Green node indicates the chemical components contained in MCXZ; Blue node refer to the targets of MCXZ; Purple node refers to the pathways involved by MCXZ putative targets; the yellow node refers to the pharmacological action of MCXZ; Red node refers to the therapeutic effect of MCXZ according to the theory of TCM.

A total of 84 compounds were identified in MCXZ, which were introduced into TCMIP V2.0 to perform target prediction, resulting in a total of 251 putative predicted targets. Detailed information regarding the identified putative MCXZ targets can be found in Supplementary Table S2. The functional enrichment analysis of the predicted MCXZ targets was performed using the KEGG database, the pharmacological effects of the pathways were determined by consulting the literature, and the relationships between pharmacological effects and traditional efficacy were analyzed. Network analysis and visualization were performed using Cytoscape software (Figure 6).

According to the functional analyses performed for the putative targets and associated pathways affected by MCXZ, combined with the known therapeutic effects of MCXZ in TCM theory, the functional effects of MCXZ could be divided into two modules. The first module includes clearing the liver and improving vision, which is associated with improving endocrine/metabolic system functions (Guan et al., 2019). The second module resolves dampness, which has anti-cancer (Chen and Wang, 2010), antidiuretic (Liu et al., 2012), and sedative (Zhang, 2016) effects. Surprisingly, both functions include anti-inflammatory, anti-microbial, immunomodulatory (Shi et al., 2019), antihyperlipidemic, hypoglycemic (Feng, 2011; Guan et al., 2019), antioxidant (Qi and Li 2018; Chen, 2020), and apoptotic effects.

### Underlying Mechanisms Through Which MCXZ Acts on DM

**FIGURE 7:**
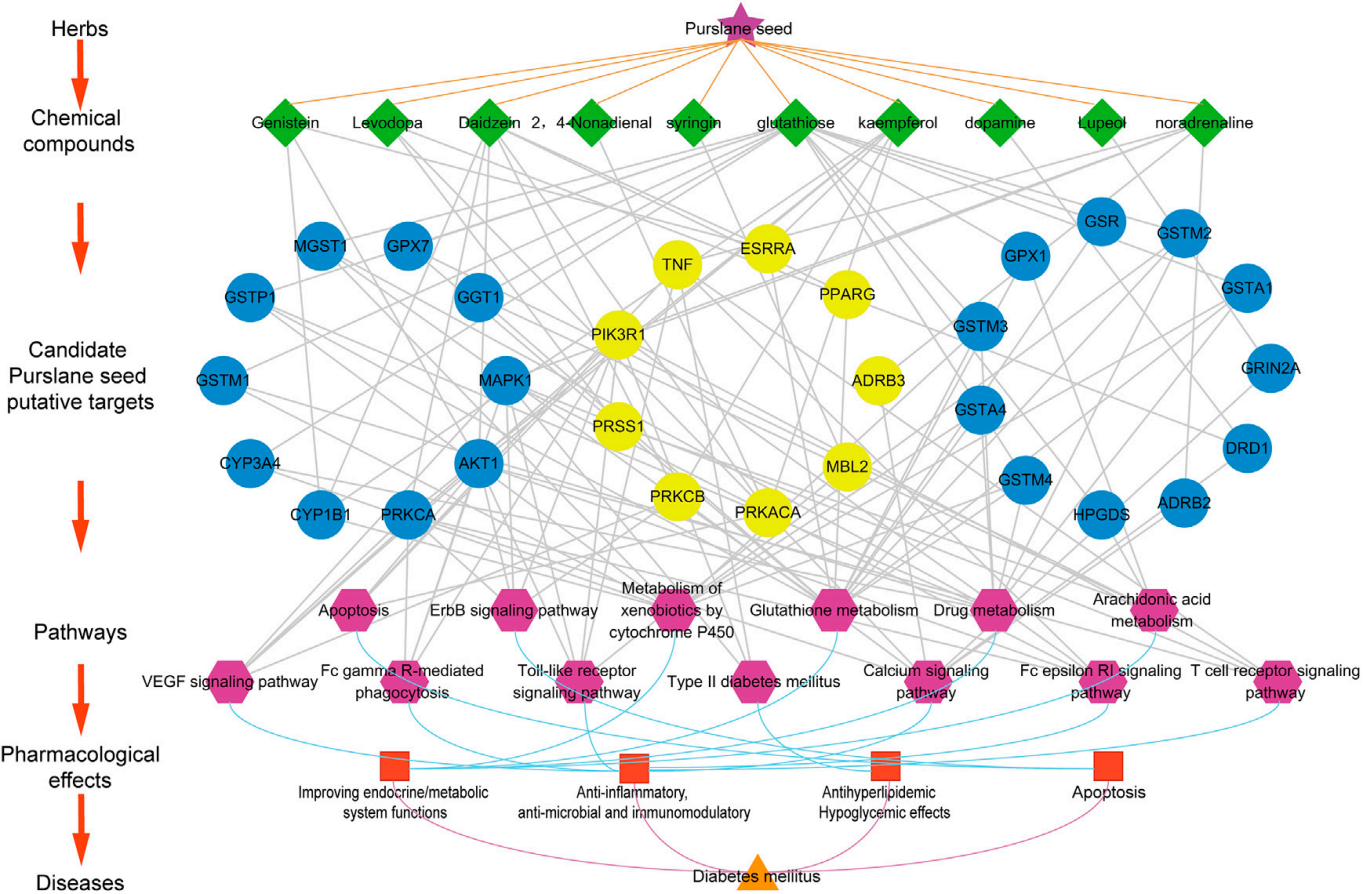
Shows the correlation among the chemical components, candidate targets, involved pathways, and corresponding pharmacological effects of MCXZ. The green node represents the chemical components contained in the MCXZ; the Blue node refers to the candidate targets of MCXZ; the Yellow node refers to the known DM-related genes; the Purple nodes refer to the regulatory pathways of MCXZ candidate targets; the Red node refers to the corresponding pharmacological effects of the main pathways of MCXZ in the treatment of DM.

According to the disease-related gene database in TCMIP V2.0, 239, the present study identified DM-related genes, shown in Supplementary Table S3. To explore the potential mechanisms through which MCXZ acts on DM, an interaction network, based on the STRING database and the putative MCXZ target-DM-related gene interactions (Figure 7) was constructed, and the topological network parameters were calculated. Supplementary Table S4 provides detailed information regarding the proposed interactions between putative MCXZ targets and DM-related genes. The network consists of 420 nodes and 4,793 edges.

To determine the hub nodes, which may have high value and perform important functions, we calculated the values of the nodes within the putative MCXZ target-DM-related gene interaction network. Consequently, 78 nodes were selected as hubs due to degree values that were greater than 2-fold the median value of all nodes in the network. Based on the direct connections between hubs, an interaction network composed of 78 hubs and 1,079 edges was established (Supplementary Table S5). After calculating the topological parameters (degree, betweenness, and closeness) of each hub, 75 major hub nodes were selected as key targets because the three topological parameters for them exceeded the corresponding median values. Among these hubs, 35 major hub nodes were known DM-related genes, of which 44 were putative targets of MCXZ and were considered to be candidate MCXZ targets for the treatment of DM. Supplementary Table S6 provides details for the 44 targets presumed to be targeted by MCXZ.

The biological functions and pathways of MCXZ target candidates for DM therapy were studied using enrichment analysis, based on the KEGG database. MCXZ appears to treat DM primarily by improving the endocrine/metabolic system, in addition to exerting anti-inflammatory, anti-microbial, immunomodulatory, antihyperlipidemic, hypoglycemic, apoptotic, and other pharmacological effects. The key DM-associated KEGG pathways involved in these pharmacological activities included Type II diabetes mellitus and inflammatory and immune-related pathways such as the Fc epsilon RI signaling pathway, Fc gamma R-mediated phagocytosis, VEGF signaling pathway, T cell receptor signaling pathway, Toll-like receptor signaling pathway, and Calcium signaling pathway. It also involved multiple endocrine/metabolic system-related pathways, including Glutathione metabolism, Metabolism of xenobiotics by cytochrome P 450, Drug metabolism, and Arachidonic acid metabolism, as well as Apoptosis-related pathways, such as ErbB signaling pathway and Apoptosis (Figure 7).

### Expression of *Akt1*, *VEGF*, *ErbB2*, and *AR* mRNA

The accuracy of network pharmacology prediction results was verified by detecting the expression levels of key genes, including *Akt1*, *VEGF*, *ErbB2,* and *AR,* which are involved in the VEGF and ErbB signaling pathway.

**FIGURE 8:**
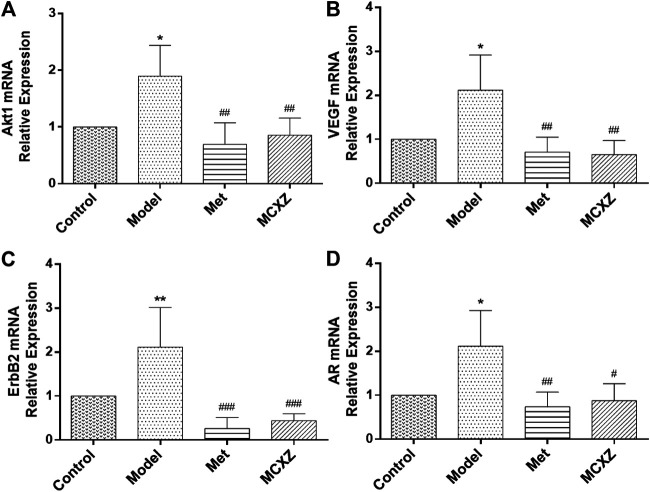
The genes expressions of *Akt1* **(A)**, *VEGF* **(B)**, *ErbB2* **(C)** and *AR* **(D)** in Control, DM, Met and MCXZ treated groups. Data are mean ± SD. ^*^ *p* < 0.05 vs. Control; ^###^ *p* < 0.001, ^##^ *p* < 0.01, ^#^ *p* < 0.05 vs. the modle group; n = 3 animals per group.

In the pancreatic tissue of diabetic model mice (model group), the expression levels of *Akt1*, *VEGF*, *ErbB2,* and *AR* mRNA were significantly increased (*p* < 0.05) compared with those in normal mice (control group). In contrast, after 4 weeks of treatment with MCXZ, the expression levels of *Akt1*, *VEGF*, *ErbB2,* and *AR* mRNA significantly decreased compared with those in the model group (*p* < 0.05, Figure 8).

## Discussion

DM has become a common and high-risk disease in modern times. Due to unhealthy lifestyles (e.g., poor diet, low physical activity, and sedentary behavior) (Mozzillo et al., 2017), patients with DM tend to be younger. According to the IDF, currently, 463 million individuals suffer from DM, a number that is expected to reach 592 million by 2035 (Liu et al., 2019). At present, the drugs used to treat DM typically aim to control blood glucose levels, and most of these drugs lack the sufficient ability to prevent and control disease symptoms, with reduced efficacy over time (Moukette et al., 2017). Therefore, researchers have begun to search for new compounds, especially among natural products, to better control blood glucose levels and associated complications.

MCXZ is widely distributed in tropical, subtropical, and temperate regions. Due to strong adaptability to changes in environmental conditions, the germplasm resources of MCXZ are relatively abundant. Studies have shown that MCXZ could be used as an effective and safe adjuvant therapy among DM subjects (El-Sayed, 2011). In this study, we found that MCXZ alone was able to directly reduce the levels of FBG and HbA1c in STZ-induced DM model mice, and the hypoglycemic effect of the low-dose MCXZ group was found to be better than the middle- and high-dose groups, which may be attributed to lower drug concentrations being more beneficial to digestion and absorption by the gastrointestinal tract.

All experimental data indicated that MCXZ induced anti-hyperglycemic effects and no histopathological damage was observed in the liver or kidney of mice after MCXZ administration, which is consistent with the literature. Chavalittumrong et al. studied the toxicity of purslane and found no histopathological damage to the brain, heart, kidney, liver, spleen, lung, kidney, or other tissues, indicating that purslane had no obvious toxic effects on the examined internal organs (Chavalittumrong et al., 2004).

To explain the material basis and molecular mechanisms of MCXZ treatment in DM, high-throughput technology (UHPLC-LTQ-Orbitrap) and the ETCM database were used to characterize the chemical components of MCXZ quickly and systematically. A total of 84 chemical components were identified, including 34 organic acids, 21 alkaloids, nine flavonoids, and seven coumarins. Some of these compounds have previously been used in the treatment of DM, such as genistein, levodopa, daidzein, 2,4-nonadienal, syringin, glutathione, kaempferol, dopamine, lupeol, and noradrenaline. Several studies have shown that genistein was able to improve dysfunctional hepatic gluconeogenesis in DM (Dkhar et al., 2018). Diabetic retinopathy is a common complication of DM, and clinical studies have also shown that the combined use of levodopa and carbidopa can reverse retinal dysfunction (Motz et al., 2020). Noradrenaline can promote insulin secretion by islet glands and regulate glucose metabolism, reducing and maintaining the stability of blood glucose levels (Zhang and Sun, 1991). Studies have shown that daidzein improved insulin resistance, dyslipidemia, and inflammation and was able to prevent DM and its related complications (Das et al., 2018). The anti-diabetic mechanism of kaempferol may be related to the ability of this substance to promote glucose metabolism and inhibit hepatic gluconeogenesis. (Alkhalidy et al., 2018). Syringin has been shown to treat DM by increasing glucose utilization and reducing plasma glucose levels in rats with insulin deficiency (Niu et al., 2008). Lupeol significantly reduced the level of blood glucose and oxidative stress in DM model rats, indicating that lupeol might have hypoglycemic activity and be useful for the treatment of DM (Malik et al., 2019).

A total of 251 putative MCXZ targets were identified using the TCMIP V2.0 database, and the relationships between the putative MCXZ targets and traditional efficacy were analyzed. The pharmacological effects of the predicted MCXZ targets and pathways are closely related to traditional efficacy. MCXZ exerts a hypoglycemic effect through the “clear the liver and improve vision” and “resolve dampness” functions, according to TCM theory, and the possible use of MCXZ in the treatment of DM was preliminarily analyzed (Ren et al., 2017). Furthermore, the interactions between potential MCXZ targets and DM targets were analyzed, and relevant pathways were identified by performing KEGG pathway enrichment analysis. A multi-level association network diagram, showing the “TCM–key active ingredients–core target–key pathways–pharmacological actions–disease effects” relationships were drawn to clarify the pharmacological basis and potential molecular mechanisms through which MCXZ exerts its effects in the treatment of DM.

After combining data from previous publications and performing a multi-dimensional network analysis, we preliminarily determined the potential pathways and targets through which MCXZ is likely to act for the treatment of DM (Figure 7). In summary, even after excluding Type II diabetes mellitus as a direct DM pathway, several pathways were identified that could indirectly affect DM and DM-associated complications. VEGF promotes angiogenesis, and an increasing number of studies have shown that VEGF plays an important role in the development of diabetic microvascular complications, such as diabetic retinopathy, skin ulcers, and kidney disease. The inhibition of VEGF expression, the prevention of VEGF receptor binding, and the inhibition of downstream signaling pathways can successfully inhibit the development of diabetic microvascular complications (Li et al., 2017; Zhou et al., 2017).

Fc gamma R-mediated phagocytosis, which is a classical immune regulatory process, may represent a key pathway for the prevention and treatment of DM. Experiments have shown that the activation of Fc gamma R-mediated phagocytosis affects the balance of glucose metabolism. Blocking Fc gamma R-mediated phagocytosis was shown to reduce DM occurrence (Feng et al., 2019).

DM has been associated with a high risk of developing cognitive impairment, and studies have shown that the Calcium signaling pathway affected gut microbiota, which improved cognitive impairment in patients with DM (Zhang et al., 2020). The primary functions of toll-like receptors include the induction of inflammatory responses and the establishment of adaptive immunity. Studies have shown that toll-like receptors play major roles in the pathogenesis of inflammation-mediated insulin resistance. Taha et al. analyzed 30 DM patients with impaired glucose tolerance and 30 healthy individuals and found a correlation between high levels of toll-like receptor four expression and DM occurrence (Taha et al., 2018).

The glutathione metabolism signaling pathway serves to present oxidative stress-induced injuries and is involved in glucose-induced insulin secretion. Increasing the glutathione concentration in plasma can improve peripheral insulin levels, reduce oxidative damage, and increase insulin sensitivity in diabetic patients (Yang, 2019). The ErbB signaling pathway functions to protect the myocardium, inhibit myocardial fibrosis, and promote angiogenesis. The activation of this pathway might be able to protect against the development of diabetic cardiomyopathy (Han et al., 2019). Other studies have shown that blocking the activation of epidermal growth factor receptor (*EGFR*) can inhibit the infiltration and oxidative stress of kidney immune cells, increase islet autophagy activity, and improve diabetic nephropathy (Li et al., 2018).

The core targets involved in these pathways include phosphoinositide-3-kinase regulatory subunit 1 (*PIK3R1*), serine protease 1 (*PRSS1*), peroxisome proliferator-activated receptor gamma (*PPARG*), protein kinase C beta (*PRKCB*), mannose-binding lectin 2 (*MBL2*), adrenoceptor beta 3 (*ADRB3*), tumor necrosis factor (*TNF*), protein kinase cAMP-activated catalytic subunit alpha (*PRKACA*), and estrogen-related receptor alpha (*ESRRA*) (Figure 7). Notably, these nine core target proteins are all known DM candidate targets, and studies have confirmed that they play key roles in relieving major DM symptoms. Studies have demonstrated a critical role for *PIK3R1* in insulin signal transduction, which is closely related to the occurrence of insulin resistance (Karadoğan et al., 2018). Studies have confirmed that activating *PPARG* could significantly improve systemic insulin sensitivity and glucose metabolism (Yang and Chan 2016). PPAR-*γ*, which is encoded by *PPARG,* is a transcription factor that can be activated by multiple ligands and is widely distributed in adipocytes and immune cells, where it has been shown to promote the differentiation of white adipocytes, increase the number of insulin receptors, promote the transcription of genes associated with insulin signal transduction, and enhance insulin signal transduction. Therefore, the effects of DM can be attenuated by activating PPAR-*γ* (Gao et al., 2016). *PRKCB* is involved in the regulation of the B cell receptor signaling pathway, apoptosis induced by oxidative stress, insulin signaling transduction, and endothelial cell proliferation (Wu et al., 2019b).

*MBL2* is a member of the lectin family, which has been associated with immune dysfunction and is commonly expressed in immune disorder-related diseases. Recently, *MBL2* has been found to play a role in the development of insulin resistance and gestational DM, and functional variations in *MBL2* can increase DM susceptibility (Muller et al., 2010). *ADRB3* is an obesity-associated gene that plays a key role in the regulation of energy balance. In many ethnic groups, the Arg64 allele in *ADRB3* is associated with the early onset of abdominal obesity and non-insulin-dependent DM (Takenaka et al., 2012). Adipose tissue is an enormously active endocrine organ that secretes various hormones and classical cytokines, such as TNF-*α* and interleukin (IL)-6. Studies have shown that the upregulation of TNF-*α* plays an important role in the induction of insulin resistance, which is associated with obesity and DM (Jaganathan et al., 2018). *PRKACA* is involved in the regulation of lipid and glucose metabolism in addition to the insulin signaling pathway (Chi et al., 2019). *ESRRA* is a key regulator of mitochondrial metabolism, able to regulate the absorption of energetic substances, the production and transport of ATP on the mitochondrial membrane, and the response of the body to energy (Dufour et al., 2007). Increased *ESRRA*-activated oxidative phosphorylation in the skeletal muscle of DM patients has been shown to improve blood glucose regulation in DM patients (Mootha et al., 2004).

To verify the accuracy of the predicted network pharmacology results, we detected the expression levels of the key genes *Akt1*, *VEGF*, *ErbB2,* and *AR,* which are members of the VEGF and ErbB signaling pathways. We found that DM upregulated the expression levels of *Akt1*, *VEGF*, *ErbB2,* and *AR* in pancreatic tissue, which is consistent with other literature (Li et al., 2019; Momeny et al., 2019; Srivastava et al., 2019; Zhang et al., 2019).

**FIGURE 9:**
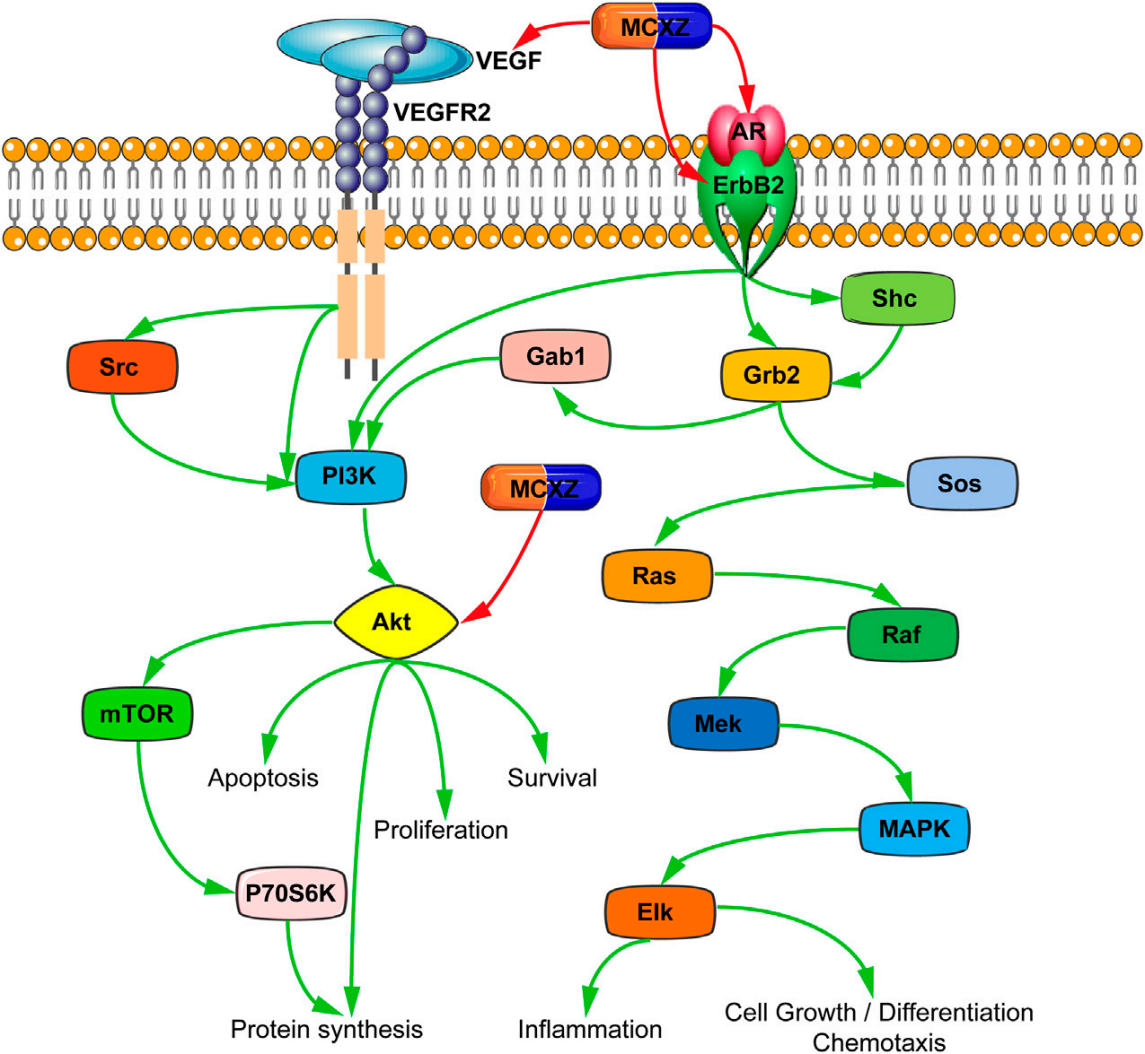
Schematic Illustration of the main pathways of MCXZ in the treatment of DM.

The results of the present study provide mechanistic insights into the effects of MCXZ in the treatment of DM (Figure 9). Our findings indicated that MCXZ might play a role in the treatment of DM in a multi-component, multi-target, and multi-pathway manner, which reflects the therapeutic characteristics of TCM.

## Conclusion

In conclusion, this study provides evidence to support MCXZ as a promising TCM agent, which can lower blood glucose levels without being associated with negative side effects. MCXZ likely acts in DM by affecting *PIK3R1*, *TNF*, *PRKACA,* and other targets associated with insulin resistance and inflammation through the actions of various chemical components, including genistein, levodopa, and daidzein, which regulate multiple pathways, such as Type II diabetes mellitus, VEGF signaling pathway, Toll-like receptor signaling pathway, and Calcium signaling pathway. These findings were consistent with the existing literature on DM, which was able to describe the molecular mechanisms through which MCXZ acts to treat DM and its complications. This study not only elucidates the effective application of TCM from a macro point of view, but the potential molecular mechanisms of action were also able to be identified from a micro perspective, combining the advantages of TCM theory with modern medical research. However, this study was based on predictions associated with existing research results, and these potential effects must be verified and confirmed through further research.

## Data Availability Statement

The raw data supporting the conclusions of this article will be made available by the authors, without undue reservation, to any qualified researcher.

## Ethics Statement

The animal study was reviewed and approved by All animal experiments were approved by the Committee on Animal Care and Use of the Institute of Chinese Materia Medica, China Academy of Chinese Medical Sciences.

## Author Contributions

JH, XZ, PW, and YQ collected the data and drafted the manuscript. LY proposed the research goal of MCXZ and provided MCXZ. JH, HX, and FL conceived of the study, participated in its design and coordination, and helped to draft the manuscript. The other authors participated in the design of the study and performed the statistical analysis. All authors read and approved the final manuscript.

## Funding

This work was supported by grants from the National Key Research and Development Program of China (2017YFC1702104, 2017YFC1702303), the National Natural Science Foundation of China (Grant No. 81830111 and 81774201), the Youth Innovation Team of Shaanxi Universities, and Shaanxi Provincial Science and Technology Department Project (No. 2016SF-378), the Fundamental Research Funds for the Central public welfare research institutes (ZXKT17058). The funding agencies had no role in the study design, the collection, analysis, or interpretation of data, the writing of the report, or the decision to submit the article for publication.

## Conflict of Interest

Author LY was employed by the Guangzhou Zhongda Pharmaceutical Development Co. Ltd.

The remaining authors declare that the research was conducted in the absence of any commercial or financial relationships that could be construed as a potential conflict of interest.
